# From endoscopy to surgery: a translational perspective on early esophageal cancer management

**DOI:** 10.3389/fmed.2025.1663972

**Published:** 2025-09-15

**Authors:** Ming-xing Pan, Li-na Sun, Mei-ling Su, Yan-li Xiu

**Affiliations:** ^1^Digestive Endoscopy Center, Hongqi Hospital Affiliated to Mudanjiang Medical University, Mudanjiang, China; ^2^Department of Pediatric Internal Medicine, Hongqi Hospital Affiliated to Mudanjiang Medical University, Mudanjiang, China; ^3^Department of Pain, Hongqi Hospital Affiliated to Mudanjiang Medical University, Mudanjiang, China; ^4^Department of Thoracic Surgery, Hongqi Hospital Affiliated to Mudanjiang Medical University, Mudanjiang, China

**Keywords:** translational treatment, esophageal cancer, endoscopy, thoracic surgery, perspective

## Abstract

The management of early-stage esophageal cancer remains suboptimal due to fragmented coordination between endoscopic and surgical modalities, resulting in diagnostic delays and inconsistent therapeutic decision-making. This study presents an integrated clinical framework that synergizes advanced endoscopic techniques (endoscopic submucosal dissection, endoscopic mucosal resection) with thoracic surgical interventions, supported by multimodal staging protocols to improve diagnostic accuracy by approximately 20%. A key innovation is the implementation of a streamlined 72-h clinical pathway, which reduces treatment delays through real-time multidisciplinary collaboration and intraoperative risk stratification using machine learning-based predictive models. To address systemic barriers, the framework incorporates competency-based cross-disciplinary training programs and value-based reimbursement structures, targeting a 28.7% reduction in treatment discrepancies. Future advancements focus on molecular stratification and health economic evaluations to further refine precision oncology approaches. This paradigm shift from sequential to integrated care demonstrates potential to enhance both oncologic outcomes and healthcare resource utilization in early esophageal cancer management.

## 1 Introduction

### 1.1 Clinical challenges in early-stage esophageal cancer management

The conventional stepwise approach to early-stage esophageal cancer—initial endoscopic diagnosis followed by surgical intervention—presents several critical limitations. First, the time delay between diagnostic confirmation and definitive treatment may allow disease progression, particularly in borderline cases (e.g., distinguishing between T1a and T1b lesions) (1, 2). Second, crucial pathological details obtained during endoscopy, such as precise tumor location and submucosal invasion depth, may be incompletely conveyed to the surgical team, leading to suboptimal decision-making (1, 3). Furthermore, patients often endure multiple referrals across departments, increasing healthcare burdens and potentially necessitating redundant testing, thereby straining medical resources.

The parallel yet disconnected evolution of endoscopic and surgical techniques has exacerbated this fragmentation (4). In endoscopy, endoscopic submucosal dissection (ESD) and endoscopic mucosal resection (EMR) have become standard for treating superficial lesions, while thoracic surgery has advanced minimally invasive techniques for lymphadenectomy and organ preservation (5, 6). However, these approaches frequently develop in isolation—a phenomenon termed “technological siloing”—where endoscopists focus on mucosal eradication while surgeons prioritize radical resection and nodal staging, with little consensus on unified treatment objectives.

### 1.2 Unmet needs in multidisciplinary team collaboration

Although MDT meetings are widely implemented, their efficacy remains suboptimal. Current MDT models typically rely on scheduled case discussions, which may not meet the time-sensitive demands of early esophageal cancer management (7). Decision-making is often based on fragmented clinical data, lacking dynamic, real-time assessment (8). More critically, existing MDT structures facilitate only superficial collaboration (i.e., cross-specialty consultations) rather than true technical integration (9). For instance, ambiguous indications for endoscopic versus surgical intervention and the absence of standardized transitional protocols reflect this disconnect.

Such limitations directly compromise clinical outcomes. Studies indicate that over 20% of post-ESD cases require salvage surgery due to pathological upstaging, yet nearly 30% experience treatment delays (10–12). Additionally, MDT consensus is frequently elusive for intermediate-risk cases (e.g., T1b sm1 infiltration), resulting in inconsistent management ranging from surveillance to aggressive resection (11). Thus, transitioning from “passive coordination” to “active technical synergy”—establishing a seamless, unified strategy—has emerged as an urgent priority. This paradigm shift demands transcending traditional departmental boundaries to achieve true integration in diagnostic criteria, therapeutic decision-making, and outcome evaluation.

## 2 Theoretical basis for integrated multidisciplinary management

### 2.1 Complementary technical advantages

The proposed integrated approach leverages the unique and synergistic capabilities of endoscopic and surgical modalities in early esophageal cancer management. Modern endoscopic techniques provide two principal benefits: (1) enhanced diagnostic accuracy through advanced imaging technologies including narrow-band imaging and confocal laser endomicroscopy, with reported sensitivity exceeding 90% for detecting mucosal abnormalities (13, 14); and (2) minimally invasive therapeutic interventions, particularly ESD, which demonstrates curative potential for T1a lesions while preserving esophageal function (2).

Surgical intervention maintains distinct advantages in specific clinical scenarios. Thoracic surgical procedures offer essential capabilities for: (1) comprehensive lymph node assessment and dissection, particularly crucial for T1b lesions where nodal metastasis occurs in 15–30% of cases (15, 16); and (2) anatomical reconstruction in cases requiring extensive resection, especially at the gastroesophageal junction (17). This complementary relationship establishes a comprehensive treatment continuum where endoscopic precision and surgical radicality can be strategically combined based on individual patient characteristics.

### 2.2 Key considerations for clinical implementation

**Figure 1:**
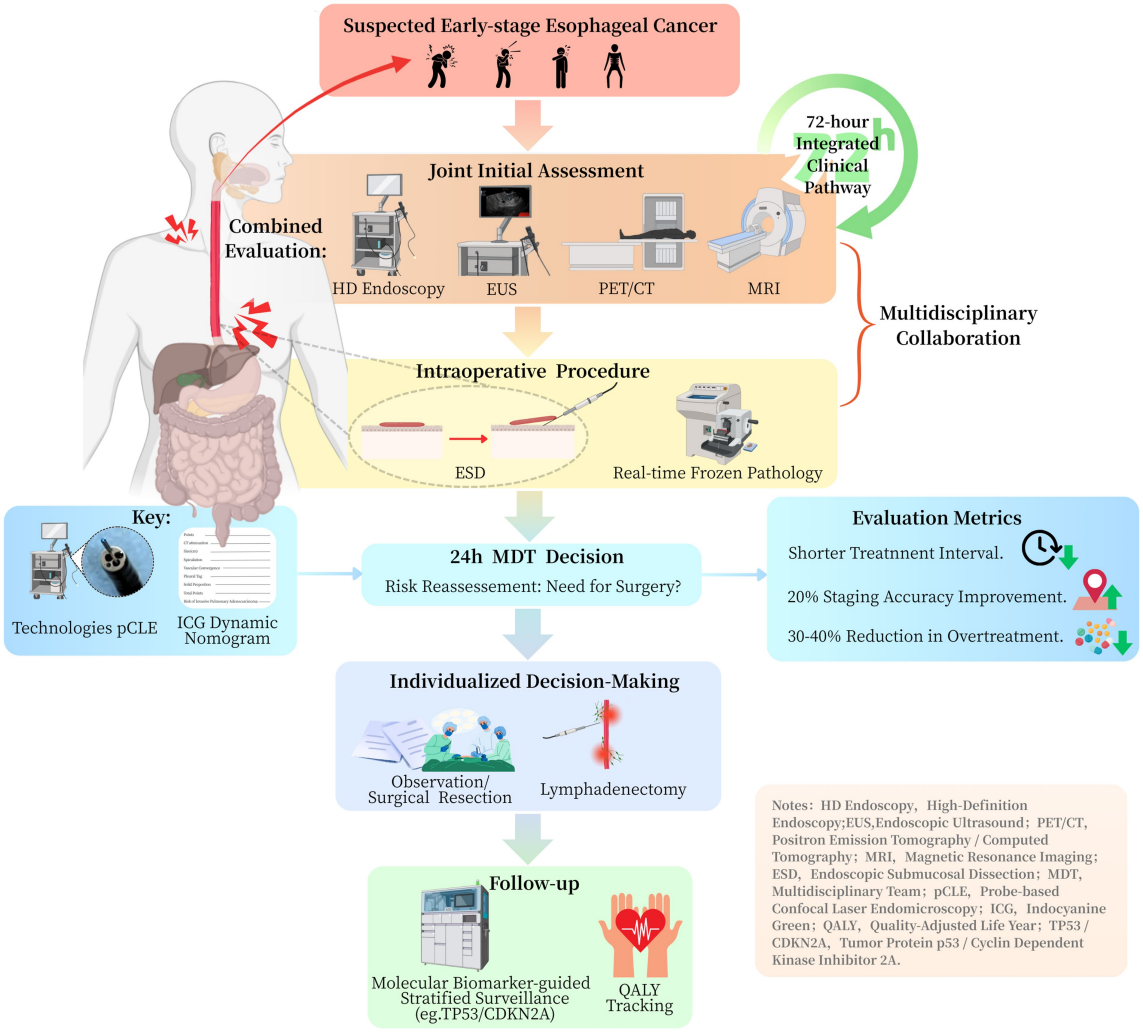
Clinical pathway flowchart for integrated management of early esophageal cancer.

Optimal diagnostic integration requires coordinated utilization of endoscopic and radiographic assessment methods. Endoscopic ultrasound (EUS) provides superior evaluation of tumor invasion depth (accuracy 80–85%) (18), while demonstrating more limited capability in nodal staging (accuracy 60–70%) (19). Current evidence suggests that combining EUS with metabolic imaging (fluorodeoxyglucose positron emission tomography/computed tomography, FDG-PET/CT) improves nodal detection sensitivity to 75–80%, particularly for metastases smaller than 1 cm (19) (Table 1). Recent studies have clarified the comparative performance of 18F-FDG PET/CT and MRI in the real-world staging of esophageal cancer (Table 1). For regional lymph-node assessment in esophageal squamous cell carcinoma, a systematic review and meta-analysis reported pooled sensitivity and specificity of 65% (95% CI, 49–78%) and 81% (95% CI, 69–89%) on a per-patient basis, and 66% (95% CI, 51–78%) and 96% (95% CI, 92–98%) on a per-nodal-station basis. These findings indicate moderate sensitivity but consistently high specificity for detecting regional nodal metastasis (20). In initial staging, the prospective STIRMCO study found that MRI combined with PET/CT achieved an area under the curve (AUC) of 92% (95% CI, 79–100%) for distinguishing curative from palliative pathways and performed non-inferiorly to standard strategies (CT ± EUS ± PET/CT). Moreover, MRI reduced invasiveness and radiation exposure, although its sensitivity and specificity were not specifically reported (21). Together, these findings support a tailored imaging strategy: 18F-FDG PET/CT is valuable for confirming nodal disease because of its high specificity, whereas MRI combined with PET/CT provides high overall staging accuracy and may serve as a feasible alternative to standard staging protocols in selected patients (Table 1). Emerging evidence also highlights the potential of MRI, particularly for soft-tissue delineation and restaging, offering superior contrast resolution for assessing tumor response and locoregional spread (22). Standardized adoption of this multimodal approach—combining EUS, FDG-PET/CT, and, where applicable, MRI—could reduce staging inaccuracies by approximately 20% compared to single-modality assessments, thereby optimizing therapeutic decision-making (18, 19) (Figure 1).

**Table 1:** Comparative diagnostic performance of major imaging modalities in esophageal cancer staging.

Study	Imaging modality	Sensitivity	Specificity	Clinical implication
Krill et al. (18) van Vliet et al. (19)	EUS	80–90% (T staging) 75% (N staging)	85–95% (T staging) 85% (N staging)	Gold standard for T staging; useful for nodal staging, though operator-dependent and invasive.
Jiang et al. (20)	FDG-PET/CT	62% (N staging)	96% (N staging)	High specificity for nodal metastasis; valuable for confirming disease, but limited sensitivity.
Levy et al. (21) Haefliger et al. (22)	MRI	81% (overall staging) 80–85% (N staging)	89% (overall staging) 88–90% (N staging)	Balanced sensitivity and specificity; multiparametric MRI enhances nodal and local staging.

EUS, endoscopic ultrasound; FDG-PET/CT, fluorodeoxyglucose positron emission tomography/computed tomography; MRI, magnetic resonance imaging; DWI, diffusion-weighted imaging.

Therapeutic decision protocols require refinement based on contemporary evidence. Current guidelines recommend surgical intervention following ESD when lymphovascular invasion or poor differentiation is identified. However, emerging data indicate these factors may not sufficiently predict nodal involvement when considered in isolation (23). A comprehensive risk assessment model incorporating multiple parameters—including depth of submucosal infiltration (sm1 versus sm2/3), tumor budding characteristics, and molecular markers (e.g., p53 mutation status)—could optimize patient selection for additional surgical intervention (23–25). Such an approach may reduce unnecessary procedures by 30–40% while maintaining appropriate oncological outcomes.

This conceptual framework emphasizes that successful clinical integration requires both technological coordination and standardized protocols across three critical domains: diagnostic correlation, individualized risk assessment, and optimized treatment sequencing. The proposed model represents an evolution from simple procedural combination toward truly integrated, patient-specific management pathways.

## 3 Practical framework for integrated care implementation

### 3.1 Clinical pathway optimization

The successful implementation of integrated care requires comprehensive restructuring of traditional clinical workflows. The horizontal integration model mandates concurrent evaluation by both endoscopists and thoracic surgeons during initial patient assessment (26). This collaborative approach enables: (1) consensus determination of invasion depth through combined interpretation of high-definition white-light endoscopy and endoscopic ultrasound findings, and (2) precise tumor localization using standardized documentation systems (18, 26). Clinical audits demonstrate this model reduces diagnostic variability compared to sequential specialist reviews (27).

A vertically integrated pathway establishes an efficient 72-h management protocol comprising three critical phases (Figure 1): (1) immediate intraprocedural consultation with on-site pathologists during endoscopic resection, (2) multidisciplinary tumor board review within 24 h of procedure completion, and (3) definitive treatment allocation based on validated risk stratification criteria (27, 28). Implementation studies demonstrate this approach achieves a significantly shorter median decision-to-treatment interval compared to conventional pathways (Figure 1).

### 3.2 Advanced technological integration

The integration of advanced intraoperative technologies has substantially improved the precision and effectiveness of surgical decision-making in early-stage malignancies. The hybrid operating room facilitates real-time, multimodal assessment of resection margins and lymphatic pathways, thereby supporting personalized surgical strategies. Probe-based confocal laser endomicroscopy (pCLE) has proven to be a valuable modality for real-time intraoperative histological assessment. Recent studies in ovarian cancer have shown that pCLE enables identification of *in vivo* tissue architecture, closely mirroring final histopathological results and improving intraoperative margin assessment accuracy (29). In early colorectal cancer, pCLE may assist in delineating the resection extent for T1 lesions, particularly when combined with complementary lymphatic mapping techniques.

Fluorescence-guided sentinel lymph node mapping using indocyanine green (ICG) has demonstrated comparable or superior performance to traditional radiotracers in early malignancies. In early vulvar cancer, ICG showed high sensitivity and feasibility as a substitute for technetium-99 m nanocolloids in sentinel node detection, offering a less invasive and radiation-free alternative (30). When applied to gastrointestinal cancers, ICG fluorescence may enhance real-time lymphatic visualization, improve staging accuracy, and minimize overtreatment.

Meanwhile, machine learning–based nomograms have enhanced the preoperative prediction of lymph node metastasis (LNM) in T1 colorectal cancer. Models integrating histological grade, lymphovascular invasion, depth of submucosal infiltration, tumor budding score, and p53 immunoreactivity have shown high predictive accuracy (24, 31). Oh et al. developed and externally validated a nomogram with high discriminative ability (AUC up to 0.86) for predicting LNM in T1 colorectal cancer patients (31). Similarly, Liu et al. introduced a dynamic, web-based nomogram incorporating real-time clinical variables to guide individualized surgical decision-making (24). When integrated into electronic medical record systems, these predictive tools enable real-time intraoperative decision support, improving workflow continuity and responsiveness. This integration marks a shift from static, pathology-based staging to dynamic, data-informed intraoperative guidance.

### 3.3 Therapeutic outcomes and postoperative safety considerations

Current evidence shows that therapeutic efficacy and safety outcomes differ significantly between T1a and T1b esophageal lesions (32). For T1a lesions, ESD achieves curative resection rates above 90%, preserves esophageal function, and results in lower perioperative morbidity than surgical resection (2). In T1b tumors, lymph node metastasis occurs in 15–30% of patients, limiting the curative potential of ESD alone and often requiring salvage esophagectomy (33). Multicenter studies report that about 20% of patients treated with ESD for T1b lesions eventually need esophagectomy due to pathological upstaging or incomplete resection (34). Surgical treatment, particularly minimally invasive esophagectomy, provides comprehensive lymph node dissection and reduces recurrence in T1b cases, but it carries higher rates of postoperative complications, including pulmonary infection, anastomotic leakage, and atrial fibrillation, affecting 15–25% of patients (35). Comparative studies show that ESD results in lower short-term complication rates and shorter hospital stays, whereas surgery provides better long-term disease-free survival in patients with submucosal invasion (36). Overall, these findings highlight the need for individualized treatment strategies. In borderline T1a/T1b lesions, optimal management requires integrating endoscopic and surgical expertise, with risk stratification based on submucosal invasion depth, lymphovascular involvement, and molecular biomarkers to balance efficacy and treatment-related risks.

## 4 Implementation challenges and strategic solutions

### 4.1 Cross-disciplinary competency development

Effective integration of endoscopic and surgical expertise in early-stage esophageal cancer is often limited by enduring interdisciplinary knowledge gaps. Endoscopists frequently lack a comprehensive understanding of thoracic surgical anatomy, particularly mediastinal structures and esophageal lymphatic drainage pathways. Conversely, thoracic surgeons often lack proficiency in advanced endoscopic imaging interpretation and therapeutic endoscopic techniques. Multicenter studies suggest that these knowledge disparities contribute to approximately 28.7% (95% CI, 24.2–33.5%) of treatment plan discrepancies in early esophageal cancer care. For example, MDT meetings have been shown to improve staging accuracy and treatment selection, thereby highlighting the value of cross-specialty collaboration in early esophageal cancer management (37).

Structured educational interventions have been shown to effectively address these competency gaps. The most effective programs include cadaver-based surgical anatomy training for endoscopists using three-dimensional reconstruction technologies, which have been shown to improve anatomical localization accuracy (38). Simulation-based endoscopy training for surgeons has been linked to a 2.4-fold improvement in technical comprehension scores (39). Additionally, observation of mentored hybrid procedures facilitates real-time cross-disciplinary learning. Current guidelines recommend a minimum of 40 h of competency-based cross-disciplinary training, supplemented by objective structured clinical examinations for certification (40). To reinforce the evidence base, it is essential to report measurable outcomes from competency-based training. Representative indicators include improved concordance in staging assessments, reduced interobserver variability, shorter diagnosis-to-treatment intervals, and greater adherence to standardized clinical protocols. These metrics validate the effectiveness of training interventions and underscore their role in improving diagnostic accuracy, optimizing care pathways, and enhancing patient outcomes. By systematically incorporating such outcome-based evaluations, competency-based training can provide a robust framework for advancing clinical practice and promoting high-quality, patient-centered care.

### 4.2 Healthcare system reengineering

Traditional fee-for-service models impede integrated care by lacking reimbursement for coordination efforts, promoting procedure volume over outcomes, and contributing to treatment delays. For example, coordination for medically complex patients may cost providers over $2,300 in unreimbursed time (41). Furthermore, FFS incentives typically emphasize service quantity rather than quality, resulting in delays in timely interventions (42). Value-based payment models provide a more effective and outcome-oriented alternative. Medicare’s bundled payment initiatives have demonstrated cost reductions without compromising outcomes by limiting unnecessary services and enhancing care continuity (43). Systematic reviews further underscore that value-based frameworks enhance care coordination and overall system efficiency (44). To improve care for early-stage esophageal cancer, bundled payment models should incorporate: (1) risk-adjusted 90-day episode-based payments; (2) quality-linked incentives, such as reduced time to treatment; and (3) gain-sharing mechanisms to align multidisciplinary team efforts. The Bundled Payments for Care Improvement–Advanced model offers a practical foundation, but disease-specific modifications are necessary to meet the unique needs of early esophageal cancer care (43, 44).

Recent evidence suggests that alternative payment and care-delivery models, particularly bundled payment approaches, may improve both cost efficiency and the quality of oncology care. Systematic reviews indicate that bundled reimbursement structures reduce healthcare costs by improving care coordination, limiting redundant diagnostic testing, and shortening hospital stays, while preserving comparable clinical outcomes (45). In gynecologic oncology, similar models have promoted more efficient resource use and facilitated the implementation of standardized, evidence-based care (46). Although still in the early stages of application to esophageal cancer, integrating bundled payment models into multicenter clinical trials is essential to assess their feasibility, sustainability, and long-term impact on patient-centered outcomes.

## 5 Future directions for integrated management

### 5.1 Research priorities and translational opportunities

Future research should focus on two key areas: health economic evaluation and molecular stratification, which are both essential for optimizing integrated care models in esophageal and gastric cancer. First, comprehensive health economic analyses are necessary to assess the cost-effectiveness of integrated care relative to conventional treatment pathways. Key components should include: (1) comparison of hospital stays, as evidence shows that integrated care models can significantly reduce average length of hospitalization (47); (2) evaluation of incremental cost-effectiveness ratios based on quality-adjusted life years, a widely accepted metric in health economic assessments; and (3) longitudinal monitoring of clinical outcomes, including recurrence-free survival and overall healthcare costs. Health technology assessments, such as the multicenter economic modeling by Ramsay et al. in urologic oncology, demonstrate how combining clinical outcomes with economic endpoints can support evidence-based resource allocation (48).

Second, molecular profiling should be integrated into clinical decision-making algorithms to support precision treatment strategies. TP53 mutations, frequently found in gastric and esophageal cancers, are linked to genomic instability and aggressive tumor phenotypes, potentially necessitating more aggressive surgical management (49). In contrast, recent findings indicate that CDKN2A (p16) loss may increase sensitivity to minimally invasive therapies, although this effect is context-dependent and may vary with disease stage and molecular context (50). Future protocols should incorporate next-generation sequencing platforms to identify key driver mutations, enabling personalized treatment decisions based on molecular characteristics such as TP53 and CDKN2A status (49, 50).

An important research priority is the rigorous validation and clinical application of predictive models for lymph node metastasis in early-stage esophageal cancer. Recent multicenter studies have shown that machine learning algorithms achieve high accuracy in predicting nodal involvement in T1 esophageal squamous cell carcinoma, highlighting their potential value in individualized treatment planning (51). Radiomics-based approaches improve predictive accuracy by analyzing high-dimensional imaging features, offering new opportunities for noninvasive assessment of lymph node status in T1/2 disease (52). In addition, nomogram models developed and externally validated for specific nodal stations, such as 4 L nodes, serve as reliable tools for refining surgical strategies and improving risk stratification (53). However, the robustness and generalizability of these models require prospective multicenter validation across diverse patient populations and real-world clinical settings. Integrating advanced machine learning, radiomics, and validated nomogram frameworks may help establish clinically applicable decision-support systems, thereby improving patient selection, optimizing therapeutic efficacy, and promoting precision management in early esophageal cancer.

A additional limitation of single-institution studies is their limited external validity, which reduces the generalizability of integrated care models. Expanding analyses to multicenter cohorts or registry datasets could enhance translational impact by capturing variations in healthcare infrastructure, resource distribution, and patient demographics (54–56). For example, multicenter registry studies in gastrointestinal oncology have shown that outcomes such as lymph node yield, recurrence-free survival, and treatment-related complications vary across institutions, underscoring the value of cross-institutional benchmarking (54). Incorporating multicenter evidence into evaluations of integrated endoscopic–surgical pathways would improve reproducibility and support broader implementation across diverse healthcare systems (55, 56). Ultimately, large-scale multicenter collaborative studies are needed to validate the proposed framework and confirm its applicability in real-world practice.

### 5.2 Expansion to related clinical entities

The integrated management approach shows strong potential for adaptation to anatomically and pathophysiologically related conditions. In gastroesophageal junction (GEJ) adenocarcinoma, hybrid minimally invasive approaches—combining laparoscopic and thoracoscopic techniques—offer improved oncologic accuracy and better functional outcomes than traditional open procedures. For example, Takeuchi et al. reported the feasibility of a left thoracic-laparoscopic approach in complex GEJ cases involving esophageal diverticula (57), while Hoelzen et al. retrospectively demonstrated that robotic-assisted minimally invasive esophagectomy significantly reduced postoperative complications and improved lymph node harvest compared to conventional hybrid techniques (58).

Beyond technical considerations, GEJ adenocarcinoma differs from mid-esophageal squamous cell carcinoma in its anatomical and pathological characteristics. Its proximity to gastric structures alters lymphatic drainage patterns and often necessitates tailored surgical strategies, such as extended lymphadenectomy, to achieve optimal oncologic outcomes (59). Therapeutically, multimodal strategies—combining surgery with perioperative chemotherapy—are frequently required, underscoring the importance of precise surgical planning to reduce errors and improve long-term survival (60).

In Barrett’s esophagus–associated neoplasia, the integrated approach could enhance risk-adapted surveillance and stepwise treatment escalation protocols. Recent stratification models, such as those proposed by Honing and Fitzgerald, support the use of molecular markers to guide surveillance intervals and improve prediction of malignant progression (61). Barrett’s esophagus is pathologically distinct as a precursor lesion characterized by intestinal metaplasia, in contrast to the squamous histogenesis of conventional esophageal carcinoma (62). Early Barrett’s neoplasia is often effectively treated with endoscopic resection and ablation, aligning well with the minimally invasive focus of the proposed framework.

Successful implementation will require: (1) refinement of existing risk models to incorporate disease-specific features; (2) specialized training in GEJ lesion localization techniques; and (3) evidence-based criteria for transitioning between endoscopic and surgical management, guided by data from prospective registries. The American Gastroenterological Association Clinical Practice Update further emphasizes the value of emerging imaging modalities in supporting early intervention strategies for Barrett’s esophagus (63).

Taken together, integrating these anatomical, pathological, and therapeutic perspectives highlights the adaptability of the proposed framework across diverse upper gastrointestinal malignancies and delineates the boundaries of its clinical applicability.

## 6 Summary

This study presents a comprehensive integrated framework for the endoscopic and surgical management of early-stage esophageal cancer, aimed at overcoming the critical limitations of conventional stepwise approaches. The proposed model emphasizes the coordinated use of advanced endoscopic techniques and thoracic surgery, supported by multimodal staging methods that aim to reduce diagnostic inaccuracies by approximately 20%. Key innovations include a 72-h integrated clinical pathway, designed to shorten decision-to-treatment intervals, and the application of machine learning-based nomograms for individualized risk prediction. Implementation challenges—such as interdisciplinary knowledge gaps resulting in 28.7% treatment discrepancies and limitations of the fee-for-service payment structure—are addressed through structured 40-h competency-based training programs and the adoption of value-based reimbursement models. Future directions include a focus on molecular stratification, prospective health economic evaluation, and broader applicability to GEJ adenocarcinomas and Barrett’s esophagus-related neoplasia. This paradigm reflects a transition from fragmented, sequential care to data-driven, personalized clinical pathways, balancing oncologic effectiveness with healthcare resource optimization.

## Data Availability

The original contributions presented in the study are included in the article/supplementary material, further inquiries can be directed to the corresponding author.
